# Global transcriptional regulator FNR regulates the pyruvate cycle and proton motive force to play a role in aminoglycosides resistance of *Edwardsiella tarda*

**DOI:** 10.3389/fmicb.2022.1003586

**Published:** 2022-09-07

**Authors:** Li-Chun Mao, Shao-Hua Li, Xuan-Xian Peng, Hui Li

**Affiliations:** ^1^State Key Laboratory of Bio-Control, School of Life Sciences, Southern Marine Science and Engineering Guangdong Laboratory (Zhuhai), Sun Yat-sen University, Guangzhou, China; ^2^Laboratory for Marine Fisheries Science and Food Production Processes, Qingdao National Laboratory for Marine Science and Technology, Qingdao, China

**Keywords:** FNR, metabolomics, alanine-aspartate-glutamate metabolism, glutamate, aminoglycoside antibiotics, the pyruvate cycle, PMF

## Abstract

Bacterial metabolism is related to resistance and susceptibility to antibiotics. Fumarate and nitrate reduction regulatory protein (FNR) is a global transcriptional regulator that regulates metabolism. However, the role of FNR in antibiotic resistance is elusive. Here, *fnr* deletion mutant was constructed and used to test the role in *Edwardsiella tarda* EIB202 (EIB202). Δ*fnr* exhibited elevated sensitivity to aminoglycosides. The mutant had a globally enhanced metabolome, with activated alanine, aspartate, and glutamate metabolism and increased abundance of glutamic acid as the most impacted pathway and crucial biomarker, respectively. Glutamate provides a source for the pyruvate cycle (the P cycle) and thereby relationship between exogenous glutamate-activated P cycle and gentamicin-mediated killing was investigated. The activated P cycle elevated proton motive force (PMF). Consistently, exogenous glutamate potentiated gentamicin-mediated killing to EIB202 as the similarity as the loss of FNR did. These findings reveal a previously unknown regulation by which FNR downregulates glutamate and in turn inactivates the P cycle, which inhibits PMF and thereby exhibits the resistance to aminoglycosides.

## Introduction

Bacterial antimicrobial resistance (AMR) poses a major threat to human and animal health around the world (Evangelista et al., 2022; Murray et al., 2022). It has been revealed that four mechanisms play a role in the resistance, including reduction of membrane permeability, activation of enzymatic degradation, elevation of efflux pumping, and down-regulation of drug-binding target (Sun et al., 2014). Clarification of these resistance mechanisms improves understanding of bacterial antibiotic resistance, but antibiotic-resistant pathogens are still out of control. Therefore, a further understanding for the mechanisms of antibiotic resistance is needed.

Recent studies have shown that bacterial metabolism is related to susceptibility and resistance to antibiotics (Lee and Collins, 2011; Peng et al., 2015a; Ye et al., 2018a; Zhang et al., 2019; Li et al., 2020), where the pyruvate cycle (the P cycle) plays a role (Su et al., 2018; Zhang et al., 2019; Li et al., 2020). The P cycle is a recently clarified metabolic pathway, which rather than the TCA cycle provides respiratory energy in bacteria (Su et al., 2018). Inactivation of the P cycle is related to antibiotic resistance (Yin et al., 2022). This is because the inactivation affects the redox state and purine biosynthesis, leading to reduction of antibiotic uptake (Ye et al., 2018b; Zhang et al., 2019; Zhao et al., 2021). On the contrary, bacteria become sensitive to antibiotics when the P cycle is activated (Li et al., 2020), especially in response to aminoglycosides antibiotics (Zhang et al., 2019). Therefore, exogenous metabolites that promote the P cycle such as alanine, glucose, and fructose can potentiate the antibiotic-mediated killing (Su et al., 2015, 2018; Peng et al., 2015b). This is because the activated P cycle elevates proton motive force, which is required for aminoglycosides uptake.

Transcriptional regulators that integrate cellular and environmental signals to control cell activity are well known in bacteria (Liao et al., 2021). Among the regulators, fumarate and nitrate reduction regulatory protein (FNR) plays a central role in bacterial oxygen response. FNR regulates multiple biological processes through monitoring environmental oxygen *via* iron–sulfur cluster assembly-disassembly in a facultative anaerobe (Crack and Le Brun, 2018). These biological processes include the switch between aerobic/anaerobic metabolism (Crack et al., 2012), nitrogen fixation (Rutten and Poole, 2019), bioluminescence (Shi et al., 2020), infection (Kuntumalla et al., 2011), proliferation (Kado et al., 2017), virulence (Barbieri et al., 2014), the structure of the gut microbiome for adapting to the gut environment (Azcarate-Peril et al., 2018), and interactions between host and pathogen (Wang et al., 2019). Most researches about FNR focus on facultative bacteria under anaerobic conditions (Lin et al., 2019; Jiang et al., 2021), but a recent study has shown that FNR may regulate antibiotic synthesis in *Serratia* in the presence of oxygen (Sun et al., 2021). However, whether the transcriptional regulator that plays a role in metabolism contributes to antibiotic resistance is elusive.

Here, the role of FNR in aminoglycoside antibiotics was investigated in an EIB202 *fnr*-deleted mutant and the underlying mechanisms are explored. EIB202 is a highly pathogenic *Edwardsiella tarda* with available genome and causes hemorrhagic septicemia in fish and gastro- and extraintestinal infections in humans (Wang et al., 2009). The bacterium has been used a bacterial model for the study of bacterial antibiotic resistance (Su et al., 2015; Peng et al., 2015b). Since FNR regulates central carbon metabolism (Kargeti and Venkatesh, 2017), which is closely related to bacterial susceptibility to aminoglycosides (Peng et al., 2015b; Zhang et al., 2019), the present study explored whether FNR plays a role in aminoglycoside resistance. The loss of *fnr* caused elevated sensitivity to aminoglycosides. This is linked to the most elevated glutamate, the most impacted alanine, aspartate, and glutamate metabolism, and activation of the P cycle in Δ*fnr*, which promoted proton motive force (PMF). Exogenous glutamate had a similar potential to *E. tarda* EIB202 (EIB202) to elevate sensitivity to aminoglycosides. These results indicate that *fnr* may regulate glutamate metabolism to play a role in the resistance to aminoglycosides in EIB202.

## Materials and methods

### Bacterial strains and culture conditions

EIB202 used in this study was obtained from professor Yuanxing Zhang, East China University of Science and Technology. EIB202 and its FNR gene deletion strain Δ*fnr* was grown at 30°C for 24 h in 50 ml Luria-Bertani (LB) broth in 250-mL flasks.

### Construction and complementation of FNR gene-deletion mutant

FNR gene deletion strain was constructed with one-step inactivation of chromosomal gene as described previously (Jiang et al., 2020). In brief, recombinant fragment was amplified from pKD13 using a pair of primer *fnr*-1F and *fnr*-1R (*fnr*-1F: AAAAGATGTTAAAATTGACCGATATCAATATTATTTAGGCAACACCTATGATTCCGGGGATCCGTCGACC, *fnr*-1R: AAAACGGCCCGCAGGCCGTCTTCTTTA TTCGCAGCGGGCGTTCATTCGCCTGTAGGCTGGAGCTGCTTCG. Sequence without underscore was *fnr* homologous sequence; Underline part was kanamycin gene homologous sequence). Then, the purified product was transformed into EIB202 component harboring pSIM6 plasmids, which express lambda red recombinase. Recombinants were selected by agar plate with 50 μg/ml kanamycin and validated by PCR amplification of kanamycin gene and *fnr* gene using two pairs of primers *Kan*-F/*Kan*-R (*Kan*-F:TGTAGGCTGGAGCTGCTT; *Kan*-R:ATTCCGGGGATCCG TCGA) and *fnr*-2F/ *fnr*-2R (*fnr*-2F: ATTCCGGAAAAACGTGTCAT; *fnr*-2R: GCA GGGCGTACGCGGATTAC), and western blotting. For gene complementation, the entire coding regions of *fnr* were amplified by PCR using primers (*fnr*-2F/ *fnr*-2R) and cloned into the modified pACYC184 plasmid. The recombinant plasmids were transformed into the Δ*fnr* and selected on Luria broth with 100 μg/ml ampicillin to construct the complemented mutant strains Δ*fnr-*resure.

### Growth curve analysis

EIB202 and its mutant Δ*fnr* were separately cultured in LB medium overnight and were diluted 1:100 (v/v) in fresh LB broth. These bacteria were cultured at 30°C with shaking 200 rpm and measured OD600 at every 2 h. All experiments were carried out in biological triplicates.

### Survival capability assay

Bacterial survival capability assay was carried out as described previously with a modification (Lin et al., 2012). To test the survival capability of bacteria in indicated antibiotic concentration, the inoculums of EIB202 and Δ*fnr* were separately cultured in 5 ml LB medium at 30°C overnight, and then the bacteria were diluted into a 5 ml fresh LB medium at a ratio of 1:1,000. Half of them contained indicated concentration antibiotic and the other was control without any antibiotic. These tubes were incubated at 30°C for 8 h. Bacterial growth was determined by measurement of OD600. The ability for survival was characterized by comparison between experimental and control groups and was termed as percent survival. At least three biologic replicates were performed.

### Metabolomic analysis

Metabolomic analysis was performed as described previously (Peng et al., 2015b). In brief, 10 ml OD600 1.0 bacteria were quenched with 60% (v/v) cold of methanol (Sigma) and centrifuged at 6,297 rcf at 4°C for 5 min, followed by extraction of metabolites using 1 ml of cold methanol. To normalize variations across samples, 10 μl ribitol (0.1 mg per ml, Sigma-Aldrich, United States) was added into each sample as an internal standard. The supernatants were concentrated drying in a rotary vacuum centrifuge device (LABCONCO). The dried extracts were then incubated with 80 μl methoxyamine hydrochloride (20 mg/ml, Sigma–Aldrich) in pyridine (Sigma–Aldrich) for 180 min at 37°C, and then metabolite derivatization was done with an identical volume of N-methyl-N(trimethylsilyl)trifluoroacetamide (Sigma–Aldrich) for another 30 min. Samples were centrifuged at 14,167 rcf for 10 min, and the supernatant was transferred into new tubes for gas chromatography–mass spectrometry (GC–MS) analysis (Agilent).

Initial peak detection and mass spectral deconvolution were performed using Agilent Technologies MSD Productivity ChemStation Software (E.02.02.1431, 2011). Metabolites were identified using spectral matching and retention indexes from the National Institute of Standards and Technology (NIST) library using the NIST MS search 2.0. After the removal of any known artificial peaks and merger of the same compounds, the internal standard allows normalization of the resulting data. The peak intensities were normalized to form a single matrix with Rt-m/z pairs (retention time-mass charge ratio pairs) for each file in the data set. Data matrix was normalized according to the internal standard (ribitol). Normalized peak intensities formed a single matrix with Rt-m/z pairs (retention time-mass charge ratio pairs) for each file in the data set. The matrix can be used for further analysis. According to a reference distribution, Z score analysis scaled each metabolite. Statistical difference was obtained by Kruskal–Wallis test and Mann–Whitney test using SPSS 13.0. A *p-*value < 0.01 was considered significant. Hierarchical clustering was completed in the R platform^1^ with the function “heatmap. 2” of “gplots library.” Multivariate statistical analysis included principal component analysis (PCA) and orthogonal partial least square discriminant analysis (OPLS-DA) implemented with SIMCA 12.0 (Umetrics, Umeå, Sweden). Control scaling was selected prior to fitting. All variables were mean-centered and scaled to Pareto variance of each variable. PCA was used to reduce the high dimension of the data set. We analyzed the differential metabolites to their respective biochemical pathways as outlined in the MetaboAnalyst 5.0.^2^ Pathways were enriched by raw *p* value <0.05. To explore a global view of bacterial metabolism of Δ*fnr*, Interactive Pathways (iPath) analysis using iPath3.0^3^ was carried out as described previously (Yamada et al., 2011).

### Real-time quantitative PCR

Total RNA samples were prepared from indicated strains using TRIzol reagent (Invitrogen). Double-stranded cDNA was synthesized from total RNA using the SYBR Perfect real-time series kits (Takara). cDNA was analyzed by real-time RT-PCR using Roche’s LightCycler 480 real-time PCR system. Quantitative PCR was performed on each cDNA sample in triplicate. 16 s rRNA was used as an internal control to normalize the expression level. The expression level was calculated using the comparative 2^–ΔΔCt^ method. The primers used for amplification are listed in Supplementary Table 1.

### Measurement of membrane potential

Measurement of membrane potential was described previously (Zhao et al., 2021). Bacteria were collected and strained with 10 μl of 3 mM DiOC2 (BacLight Bacterial Membrane Potential Kit, Invitrogen) for 30 min at 30°C. Aliquots with 1 ml of culture were added into flow tubes before analysis. Samples were analyzed on a FACSCalibur flow cytometer (Becton Dickinson, San Jose, CA, United States) at 30°C. Each sample was observed with forwarding versus side scatter and gated before the acquisition of data. Settings were optimized according to the manual. The computational formula of membrane potential: Log[10^3/2^ × (red fluorescence/green fluorescence)]. Experiments were repeated at least in three independent biological replicates.

### Measurement of enzyme activity

Measurement of enzyme activity was performed as previously described with a few modifications (Jiang et al., 2020). A volume of 30 ml bacterial cultures at OD600 = 1.0 were collected by centrifugation. After washing, bacterial cells were crushed by ultrasonic crusher for 10 min at a 200-W on ice. Supernatants containing 200 μg of total proteins were transferred to a pyruvate dehydrogenase (PDH) reaction mix (0.15 mM MTT, 1 mM MgCl_2_, 0.5 mM PMS, 0.2 mM TPP, 2 mM sodium pyruvate, and 50 mM PBS), an α-ketoglutarate dehydrogenase (KGDH) reaction mix (0.15 mM MTT, 1 mM MgCl_2_, 0.5 mM PMS, 0.2 mM TPP, 50 mM α-ketoglutaric acid potassium salt, and 50 mM PBS), a succinate dehydrogenase (SDH) reaction mix (0.15 mM MTT, 1 mM PMS, 5 mM sodium succinate, and 50 mM PBS), and a malate dehydrogenase (MDH) reaction mix (0.15 mM MTT, 1 mM PMS, 50 mM PBS, and 50 mM malate) to a final volume of 200 μl in a 96-well plate. Subsequently, the plate was incubated at 30°C for 30 min for PDH/KGDH/MDH activity and at 30°C for 5 min for SDH activity and then measured at 566 nm for colorimetric readings. The plates were protected from light during the incubation. Experiments were repeated at least four independent biological replicates.

### Bactericidal assay

Bactericidal assay was carried out as described previously (Allison et al., 2011; Peng et al., 2015b). After cultured at 30°C for 24 h, bacterial cells were collected by centrifugation at 6,297 rcf for 5 min. The samples were washed with sterile saline three times and then resuspended in M9 minimal media containing 10 mM acetate, 1 mM MgSO_4_, and 100 μM CaCl_2_ to arrive at OD600 0.2. Aliquot of 5 ml bacterial suspension with desired glutamate or/and gentamicin was incubated at 30°C and 200 rpm for 6 h. To determine bacterial count, 100 μl of cultures were obtained and then serially diluted. An aliquot of 5 μl of each dilution was plated on LB agar.

## Results

### Resistance of Δ*fnr* to aminoglycoside antibiotics

**Figure 1:**
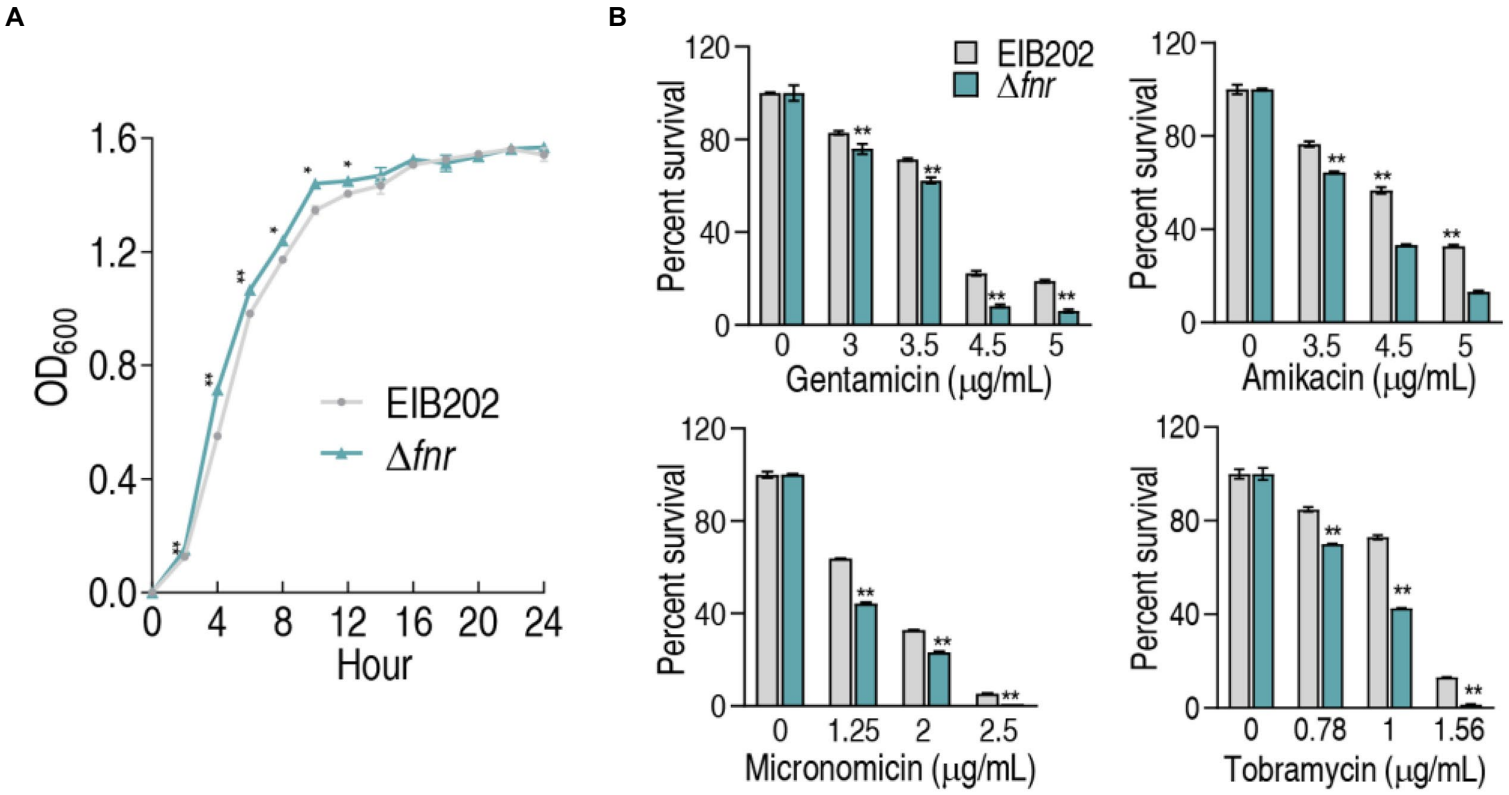
Growth and survival of genetically modified strains of *fnr* deletion. **(A)** Growth curve of EIB202 and Δ*fnr*. **(B)** Survival of Δ*fnr* and its parent strain EIB202 to aminoglycoside antibiotics. Results are displayed as means ± SEM, and significant differences are identified (*p < 0.05; **p < 0.01) as determined by Student’s *t*-test. At least three biological repeats were carried out.

To understand the role of FNR in resistance to aminoglycoside antibiotics, *fnr* deletion mutant was constructed (Supplementary Figure 1). First, the growth curve of EIB202 and Δ*fnr* was detected, showing that the loss of *fnr* caused a higher growth during 2–12 h but not later (Figure 1A). Then, survival capability assay was used to test bacterial sensitivity to aminoglycosides gentamicin, micronomicin, tobramycin, and amikacin. The loss of *fnr* caused elevated sensitivity to these antibiotics (Figure 1B). These results indicate that the absence of *fnr* increases bacterial sensitivity to aminoglycoside antibiotics.

### Metabolomic profiling of Δ*fnr*

Reports have shown that bacterial metabolomes contribute to antibiotic efficacy (Peng et al., 2015b; Zhao et al., 2021), and gene-deleted mutants exhibit differential metabolomes (Jiang et al., 2020). Therefore, GC–MS-based metabolomics was used to investigate whether the loss of *fnr* affects metabolic profiles. Four biological replicates with two technical repeats in each group were performed, yielding a total of 16 data sets. The high reproducibility of the identification in the discovery phase is shown in (Supplementary Figure 2A). This led to identification of 74 metabolites each sample (Supplementary Figure 2B). Biological categories of the identified metabolites were searched against the Kyoto Encyclopedia of Genes and Genomes (KEGG). The categories showed that 29.72% (22), 27.02% (20), 24.32% (18), 10.81% (8), and 8.10% (6) of metabolites belong to carbohydrate, amino acid, fatty acid, nucleotide, and other, respectively (Supplementary Figure 2C).

**Figure 2:**
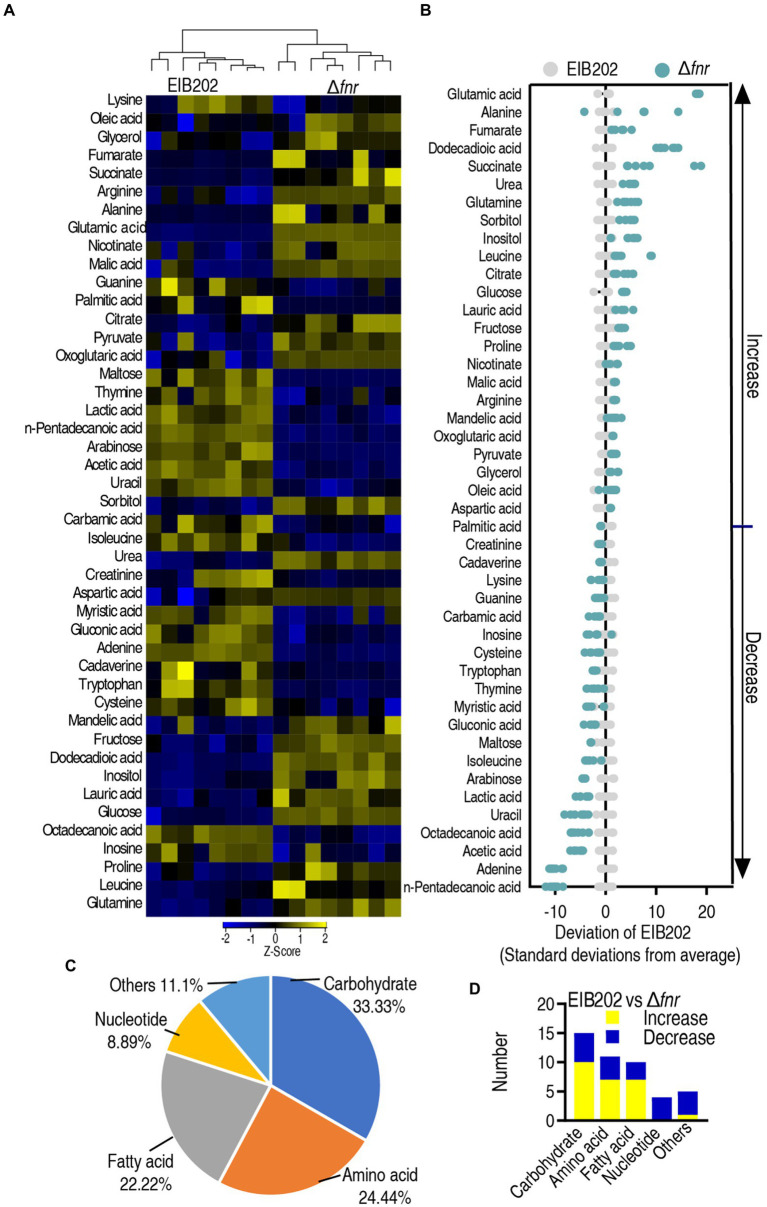
Differential abundance of metabolites in EIB202 and Δ*fnr*. **(A)** Heat map showing relative abundance of different metabolites (row). Heat map scale (blue to yellow, low to high abundance) is shown below data. **(B)** Z score plots of differential abundances of metabolites based on control. Data from tested groups are separately scaled to the mean and standard deviation of the control (EIB202). Each point represents one metabolite in one technical repeat and is colored by sample type (gray, control; green, Δ*fnr*). **(C)** Category of detected different metabolites in Δ*fnr*. **(D)** The number of changed metabolites in each category of Δ*fnr* and EIB202.

Compared with the metabolome of EIB202, 45 (60.8%) metabolites showed differential abundances (*p* < 0.05) in Δ*fnr* (Figure 2A). *Z*-value based on the control group was calculated, showing that it spanned from −10.23 to 18.17 in Δ*fnr* (Figure 2B). We further examined the metabolic categories of these differential metabolites. They showed differential percentages with a ranking of carbohydrate > amino acid > fatty acid > other > nucleotide in Δ*fnr* (Figure 2C). The numbers of up- and down-regulated metabolites in these categories were listed in (Figure 2D). These results indicate that the loss of *fnr* affects bacterial metabolic profile.

### Enrichment of metabolic pathways involved in Δ*fnr*

**Figure 3:**
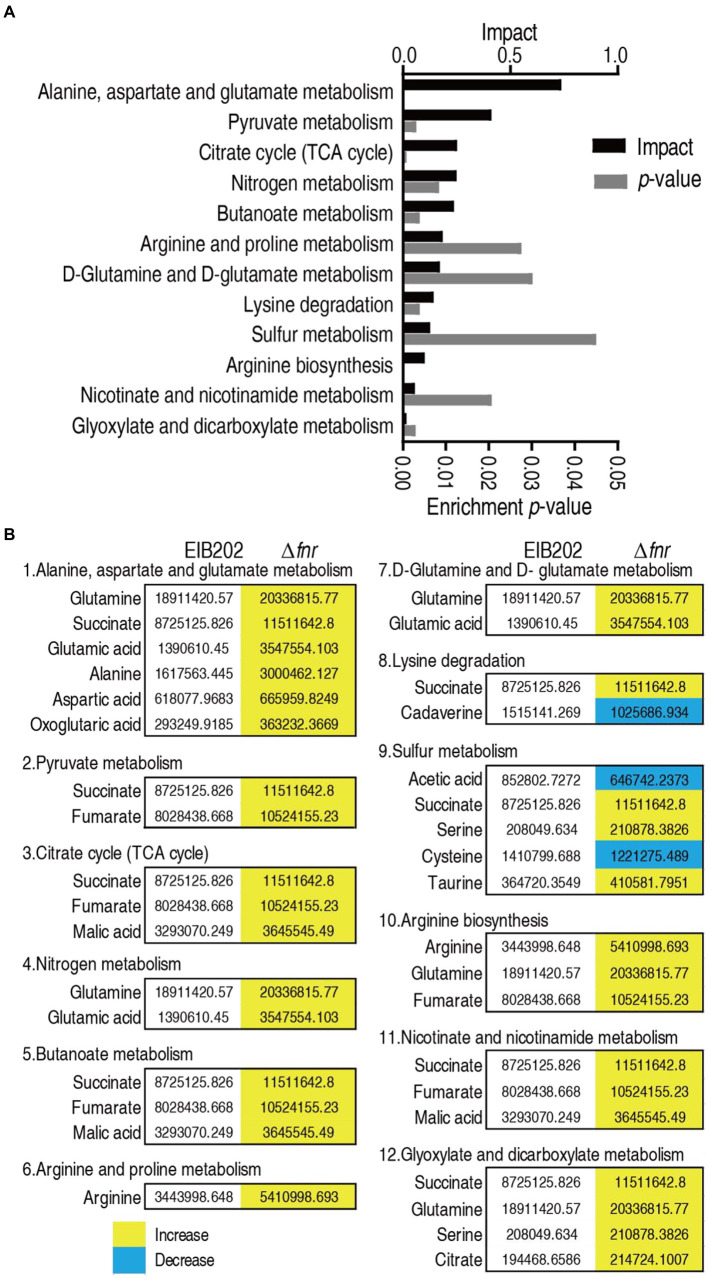
Pathway enrichment. **(A)** Pathway enrichment of varied metabolites in Δ*fnr*. **(B)** Integrative analysis of metabolites in significantly enriched pathways. Yellow color and green color indicate increased and decreased metabolites, respectively.

Enriched metabolic pathways are important for understanding the metabolome alteration due to the loss of *fnr*. A total of 12 pathways were enriched after analyzing differential metabolites between Δ*fnr* and EIB202. The 12 pathways were alanine, aspartate, and glutamate metabolism; pyruvate metabolism; TCA cycle; nitrogen metabolism; butanoate metabolism; arginine and proline metabolism; D-glutamine and D-glutamate metabolism; lysine degradation, sulfur metabolism; arginine biosynthesis; nicotinate and nicotinamide metabolism; and glyoxylate and dicarboxylate metabolism (Figure 3A). Except for lysine degradation and sulfur metabolism, in which part metabolites detected were increased or decreased in abundance, the other pathways showed that all metabolites were increased in abundance (Figure 3B). Notably, the top three enriched metabolic pathways are closely interrelated. Specifically, parts of pyruvate metabolism (pyruvate → acetyl-CoA and → oxaloacetate) plus the TCA cycle belong to the P cycle (Su et al., 2018), while alanine, aspartate and glutamate metabolism provides fuels for the P cycle.

### Identification of crucial biomarkers using multivariate data analysis

**Figure 4:**
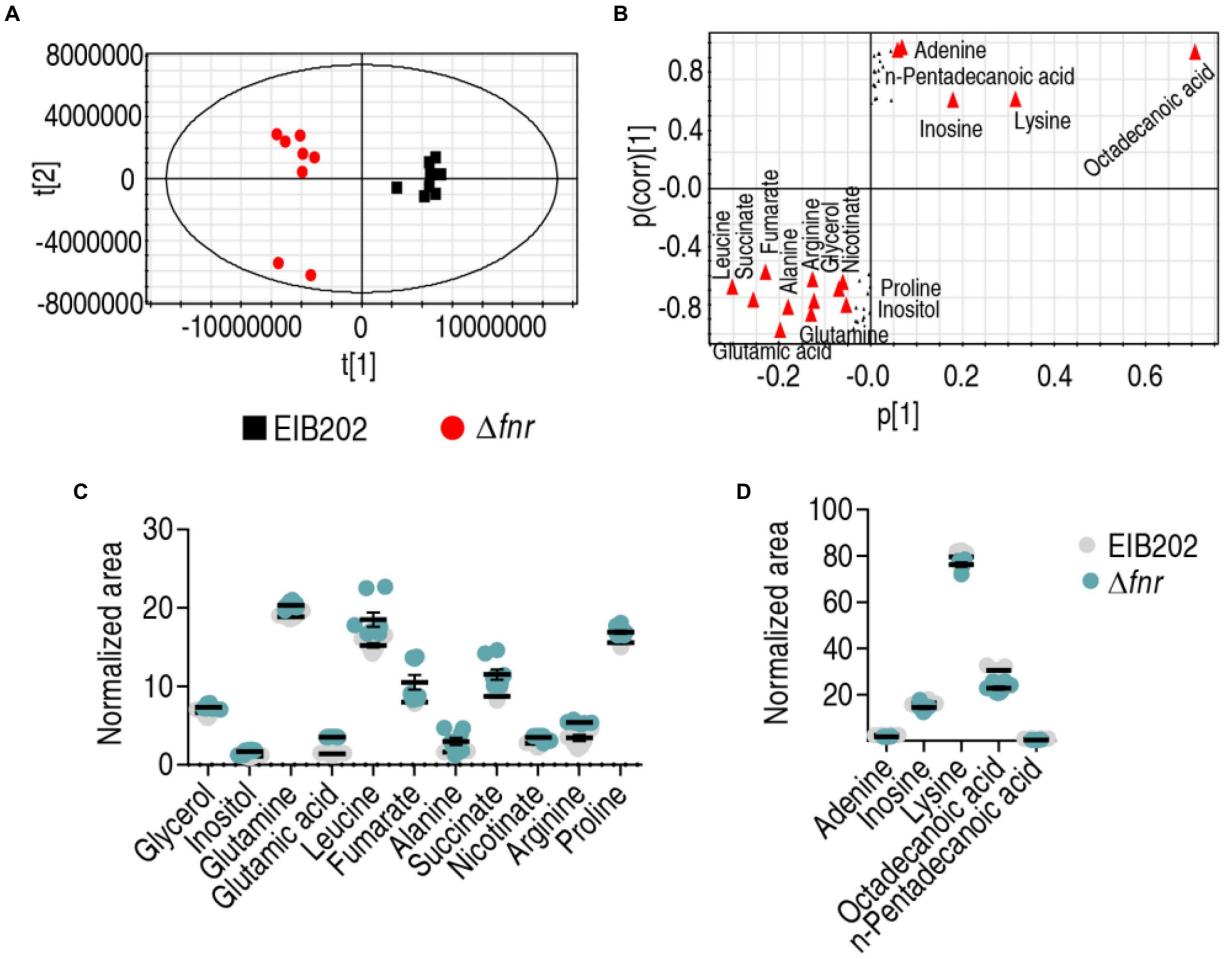
Identification of crucial metabolites. **(A)** The PCA analysis of EIB202 and Δ*fnr*. Each dot represents the technical replicate of samples in the plot. **(B)** S-plot generated from OPLS-DA. Predictive component p[1] and correlation p(corr)[1] differentiate Δ*fnr* from EIB202. The triangle represents metabolites in which candidate biomarkers are marked. **(C)** and **(D)** Scatter diagram of 16 biomarkers.

To identify crucial biomarkers representing differential metabolomes in Δ*fnr*, orthogonal partial least-squares discriminant analysis (OPLS-DA) was applied for the recognition of the sample patterns, followed by ranking the altered abundance of metabolites in loading. Δ*fnr* was separated from the control group EIB202 by predictive component [1] (Figure 4A). Discriminating variables were present with S-plot when cut-off values were set as greater or equal to 0.05 and 0.5 for the absolute value of covariance *p* and correlation *p* (corr), respectively. Glutamic acid, leucine, succinate, fumarate, alanine, arginine, glutamine, glycerol, nicotinate, inositol, proline, adenine, pentadecanoic acid, inosine, lysine, and octadecanoic acid are identified as biomarkers in the predictive component [1] between the mutant and control (Figure 4B). Among the 16 metabolites, 11 (glycerol, inositol, glutamine, glutamic acid, leucine, fumarate, alanine, succinate, nicotinate, arginine, proline) were increased and 5 (adenine, inosine, lysine, octadecanoic acid, and pentadecanoic acid) were decreased (Figures 4C,D). Out of these metabolites, glutamic acid belongs to alanine, aspartate, and glutamate metabolism, the biggest impact pathway. Glutamic acid has the most absolute value of covariance *p* and the most difference in abundance between EIB202 and Δ*fnr.* Therefore, glutamic acid is identified as the most crucial biomarker.

### iPath analysis

**Figure 5:**
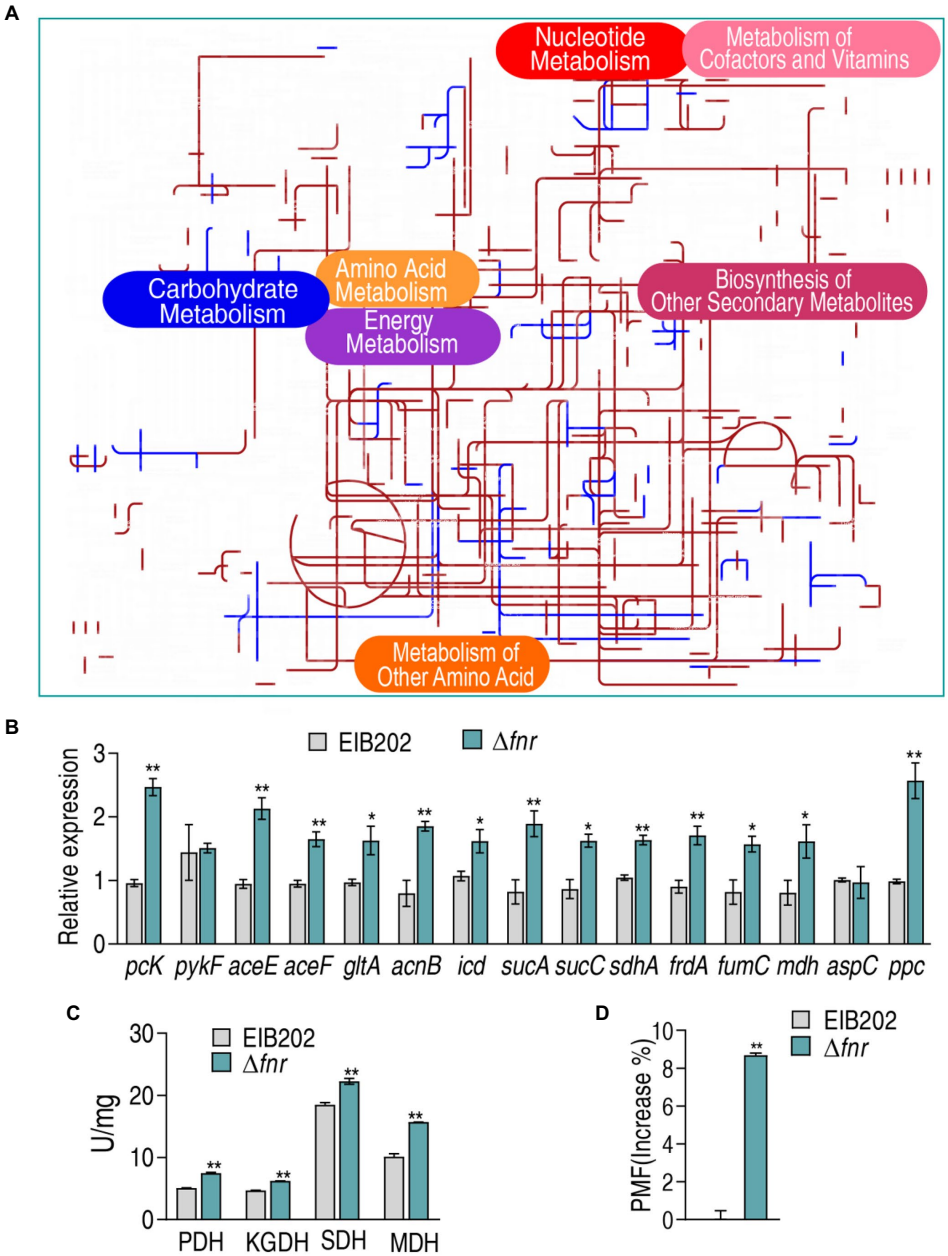
Comparative metabolic pathway analysis and enzyme activity measurement between Δ*fnr* and its parent strain EIB202. **(A)** Analysis of the metabolic profiles resulting from Δ*fnr* provides a better insight into the effects of 45 significant metabolites (*p* < 0.05). Based on the KEGG compound (http://www.kegg.jp/kegg/compound/), metabolic network pathways in Δ*fnr* are further analyzed with iPath3.0 (https://pathways.embl.de/). Purple line represents increase and blue line represents decrease in Δ*fnr*. **(B)** qRT-PCR for expression of pyruvate cycle genes. **(C)** Activity of PDH, KGDH, SDH and MDH in EIB202. **(D)** Membrane potential of EIB202 and Δ*fnr*. Results are displayed as means ± SEM, and significant differences are identified (*p < 0.05; **p < 0.01) as determined by Student’s *t*-test. At least three biological repeats were carried out.

Comparative metabolic pathway analysis between Δ*fnr* and EIB202 was carried out in iPath. The resulting global overview map provides a better insight into the effects of the *fnr* loss, where brown line represents increased pathways and blue line represents decreased pathways in Δ*fnr* (Figure 5A). TCA cycle and energy metabolism were elevated (Figure 5A), implying the importance of the activation of the pyruvate cycle since the pyruvate rather than the TCA cycle provides respiratory energy (Su et al., 2018). To validate the contributing role of the pyruvate cycle in Δ*fnr*, expression of 15 genes and activity of four key enzymes in the pyruvate cycle were measured. The four key enzymes include PDH that transforms pyruvate into acetyl-CoA, KGDH that converts a-ketoglutarate to succinyl-CoA, SDH that catalyzes the oxidation of succinate to fumarate, and MDH that catalyzes the interconversion of L-malate and oxaloacetate. Among the 15 genes, 13 were elevated and the others were not changed in Δ*fnr* (Figure 5B). Consistently, activity of the four enzymes was elevated due to the loss of *fnr* (Figure 5C). Therefore, proton motive force (PMF) was promoted (Figure 5D). These results indicate that the loss of *fnr* promotes the pyruvate cycle to elevate PMF.

### Exogenous glutamate promotes gentamicin-mediated killing to EIB202

**Figure 6:**
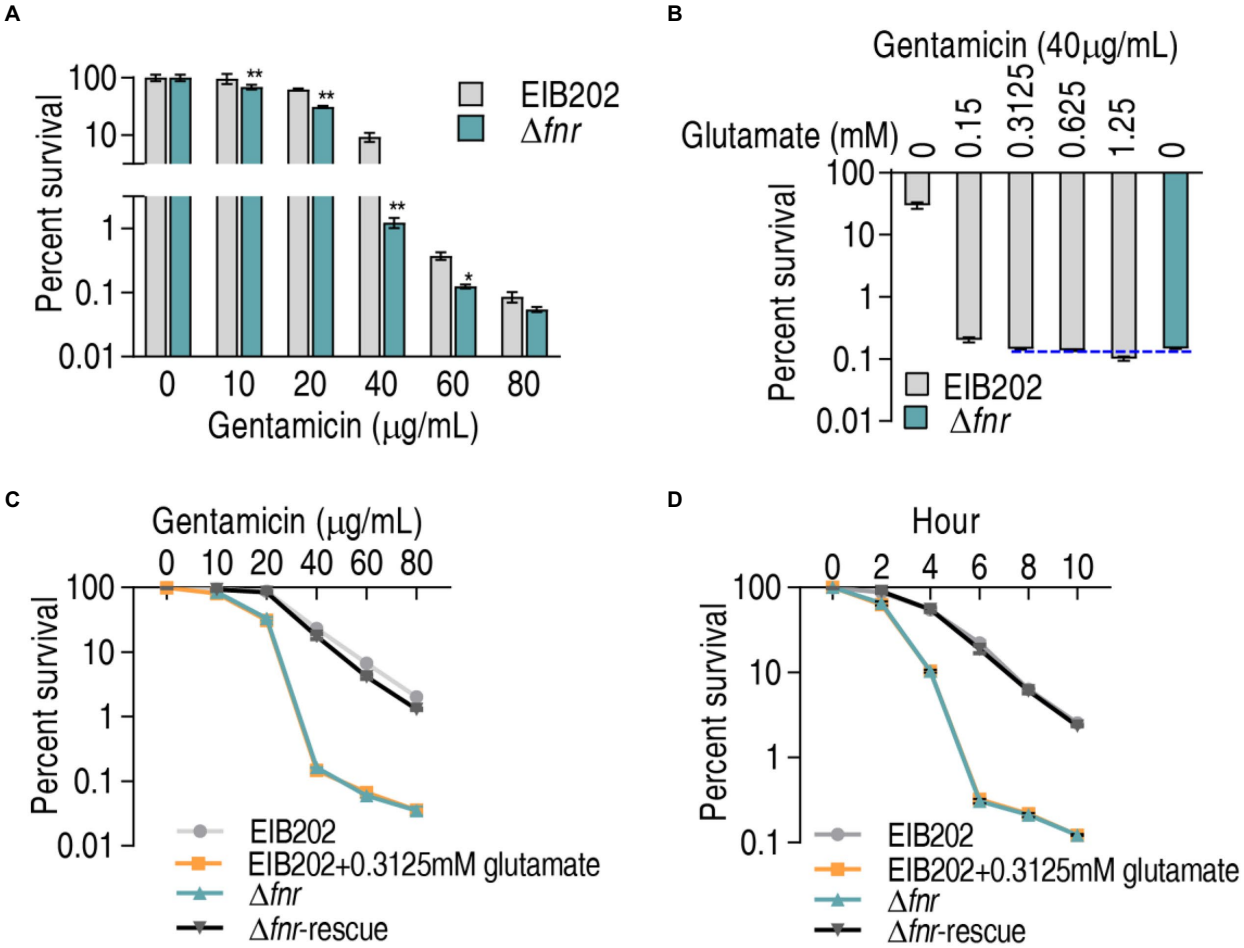
Exogenous glutamate elevates sensitivity of EIB202 to gentamicin. **(A)** Percent survival of EIB202 and Δ*fnr* in the indicated concentration of gentamicin. **(B)** Percent survival of EIB202 and Δ*fnr* in the indicated concentration of glutamate plus 40 μg/ml gentamicin. **(C)** Percent survival of EIB202 and Δ*fnr* in the indicated concentration of gentamicin plus 0.3125 mM glutamate. **(D)** Percent survival of EIB202 and Δ*fnr* in the indicated times with 0.3125 mM glutamate plus 40 μg/ml gentamicin. Results are displayed as means ± SEM, and significant differences are identified (*p < 0.05; **p < 0.01) as determined by Student’s *t*-test. At least three biological repeats were carried out.

Glutamate is linked to the P cycle and thereby the most elevated glutamate can fuel the P cycle, which is responsible for the elevated sensitivity to aminoglycosides in Δ*fnr*. Logically, when glutamate is added to a certain extent, similar sensitivity to the antibiotics may be obtained in EIB202. To explore this idea, viability of EIB202 and Δ*fnr* was measured in different doses of gentamicin using bactericide assay. Lower survival was detected in Δ*fnr* than EIB202 in a gentamicin dose-dependent manner (Figure 6A). However, the difference of the survival between Δ*fnr* and EIB202 disappeared with the increasing glutamate concentrations. Specifically, survival was lower in Δ*fnr* than EIB202 in medium with 0.15 mM glutamate, but similar and higher viability was detected in medium with 0.3125 mM and 0.625–1.25 mM glutamate, respectively in EIB202 than Δ*fnr* without gluamate (Figure 6B). Moreover, when 0.3125 mM glumate was supplemented, an increased sensitivity in EIB202 was detected in a manner of gentamicin-dependence and time-dependence, which was the same as Δ*fnr* did without glutamate (Figures 6C,D). These results indicate that the elevated glutamate may be a reason why Δ*fnr* is sensitive to aminoglycosides.

### Exogenous glutamate promotes gentamicin-mediated killing to EIB202 *via* activating the pyruvate cycle

**Figure 7:**
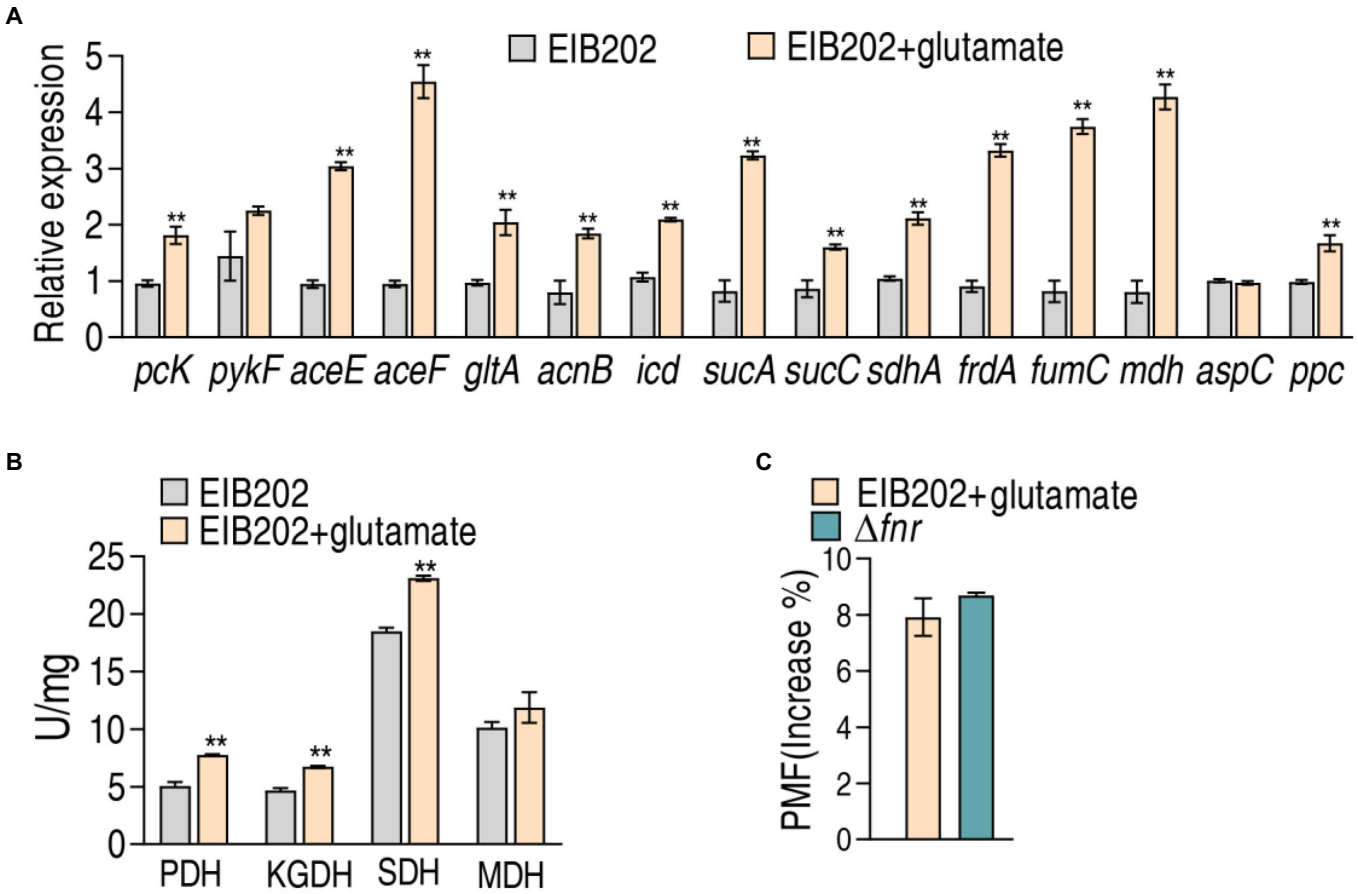
Effect of exogenous glutamate on gene expression, enzyme activity, and PMF in EIB202. **(A)** qRT-PCR for expression of genes of the P cycle in EIB202 with or without 0.3125 mM glutamate. **(B)** Activity of enzymes of the P cycle in EIB202 with or without 0.3125 mM glutamate. **(C)** Measurement of PMF in EIB202 with 0.3125 mM glutamate and Δ*fnr*. Results are displayed as means ± SEM, and significant differences are identified (**p < 0.01) as determined by Student’s *t*-test. At least three biological repeats were carried out.

Further experiments were focused whether exogenous glutamate promotes the pyruvate cycle for more PMF to elevate the sensitivity to gentamycin in EIB202 as Δ*fnr* did. To test this, qRT-PCR was used to quantify the gene expression of the pyruvate cycle in EIB202. Among the detected 15 genes, 13 were increased when exogenous glutamate was added (Figure 7A). Consistently, exogenous glutamate promoted activity of PDH, KGDH, and SDH, which led to the similar PMF as Δ*fnr* did (Figures 7B,C). These results indicate that the elevated glutamate is a reason why Δ*fnr* is sensitive to aminoglycosides.

## Discussion

It has been documented that bacterial metabolic environment confounds antibiotic susceptibility (Peng et al., 2015b; Zhao et al., 2021), but the role of global transcriptional regulator FNR that regulates oxygen response in antibiotic resistance is largely unknown. The present study uses EIB202, a model *E. tarda* that causes a great loss in aquaculture, to explore whether FNR plays a role in the resistance aminoglycosides. To do this, *fnr* deletion mutant was constructed to test sensitivity to aminoglycoside antibiotics. The absence of *fnr* causes elevated sensitivity to the four types of aminoglycoside antibiotics tested. For understanding whether *fnr* mediates the resistance *via* managing metabolism, GC–MS-based metabolomics is used to characterize the altered metabolic profile caused by the loss of *fnr*. The loss leads to an activated metabolome, characterizing the most elevated glutamate and the most impactful alanine, aspartate, and glutamate metabolism as the most consequence of the *fnr* absence. The dominantly enhanced metabolome and the most elevated glutamate and impactful metabolic pathway suggest that the P cycle plays a crucial role in the resistance. This is demonstrated by elevated gene expression, enzymatic activity, and PMF, which supports the conclusion on the PMF-potentiated killing of aminoglycoside antibiotics. It proceeds by increasing glutamate to promote the P cycle and thereby elevate PMF. These results indicate that FNR plays a role in the aminoglycosides resistance *via* metabolic regulation. This is first time to reveal an FNR-related antibiotic resistance mechanism.

FNR regulates metabolism has been reported (Crack and Le Brun, 2018; Jiang et al., 2021), but information regarding its influence on metabolic profile is not available. The present study shows that the loss of *fnr* causes globally metabolic activation, indicating that the global transcriptional regulator depresses global metabolism. This may be related to its role in regulating respiratory metabolism that produces the energy and substrate molecules to influence other metabolisms. In bacteria, the P cycle instead of the TCA cycle provides respiratory energy (Su et al., 2018). Thus, the present study demonstrates that the loss of FNR leads to the elevation of gene expression and enzymatic activity of the P cycle and then shows that the resulting PMF contributes to the sensitivity to gentamicin. These results not only provide a solid proof that FNR regulates global metabolism, but also reveal that the regulation contributes to aminoglycosides resistance.

Another interesting finding is that the loss of *fnr* elevates glutamate, which, in turn, increases the sensitivity to aminoglycosides. The finding is consistent with the reports that reduced glutamate is a characteristic feature in kanamycin-resistant *E. tarda* and complemented glutamate potentiates kanamycin-mediated killing (Peng et al., 2015b; Su et al., 2018). The present study demonstrates that exogenous glutamate promotes bacterial sensitivity to gentamicin in a dose-dependent manner in EIB202. The resulting effect can be equal to that caused by the absence of *fnr*, which supports the conclusion that FNR regulates resistance to gentamicin through modulation of glutamate level. Furthermore, the modulation influences the P cycle and PMF. These results indicate that the regulation of glutamate is a mechanism by which FNR plays a role in aminoglycosides resistance.

## Conclusion

An *fnr*-deleted mutant is constructed to explore a role of the gene in aminoglycoside antibiotics. The mutant shows an elevated sensitivity to gentamicin, micronomicin, tobramycin, and amikacin, which is linked to the most activated alanine, aspartate, and glutamate metabolism and the P cycle and the most elevated glutamic acid. These promote PMF that contributes to aminoglycosides-mediated killing. Further evidence show that exogenous glutamate plays the same role as *fnr* deletion, including activation of the P cycle, promotion of PMF, and elevation of gentamicin-mediated killing. Therefore, FNR plays a role in aminoglycoside resistance by modulation of glutamate metabolism. These findings reveal the underlying mechanism by which FNR regulates aminoglycoside antibiotic resistance.

## Data availability statement

The original contributions presented in the study are included in the article/Supplementary material, further inquiries can be directed to the corresponding author.

## Author contributions

HL conceptualized the project and designed the protocol. LM and SL performed the experiments and interpreted the data. HL and XP wrote the manuscript. All authors contributed to the article and approved the submitted version.

## Funding

This work was sponsored by the National Natural Science Foundation of China (31930115 and 31902414), International Exchanges Scheme (NSFC-RS; 32061133007), Innovation Group Project of Southern Marine Science and Engineering Guangdong Laboratory (Zhuhai; no. 311020006).

## Conflict of interest

The authors declare that the research was conducted in the absence of any commercial or financial relationships that could be construed as a potential conflict of interest.

## Publisher’s note

All claims expressed in this article are solely those of the authors and do not necessarily represent those of their affiliated organizations, or those of the publisher, the editors and the reviewers. Any product that may be evaluated in this article, or claim that may be made by its manufacturer, is not guaranteed or endorsed by the publisher.

## Supplementary material

The Supplementary material for this article can be found online at: https://www.frontiersin.org/articles/10.3389/fmicb.2022.1003586/full#supplementary-material

## Footnotes

^1^https://cran.r-project.org/ (Accessed August 20, 2022).

^2^http://www.metaboanalyst.ca/ (Accessed August 20, 2022).

^3^https://pathways.embl.de/ (Accessed August 20, 2022).
