# Supplementary material for: Global transcriptional regulator FNR regulates the pyruvate cycle and proton motive force to play a role in aminoglycosides resistance of Edwardsiella tarda
Source: Front Microbiol. 2022 Sep 7;13:1003586. doi: 10.3389/fmicb.2022.1003586 (PMC9490114; doi:10.3389/fmicb.2022.1003586)
Supplement: Supplementary file 1 [file Data_Sheet_1.docx]

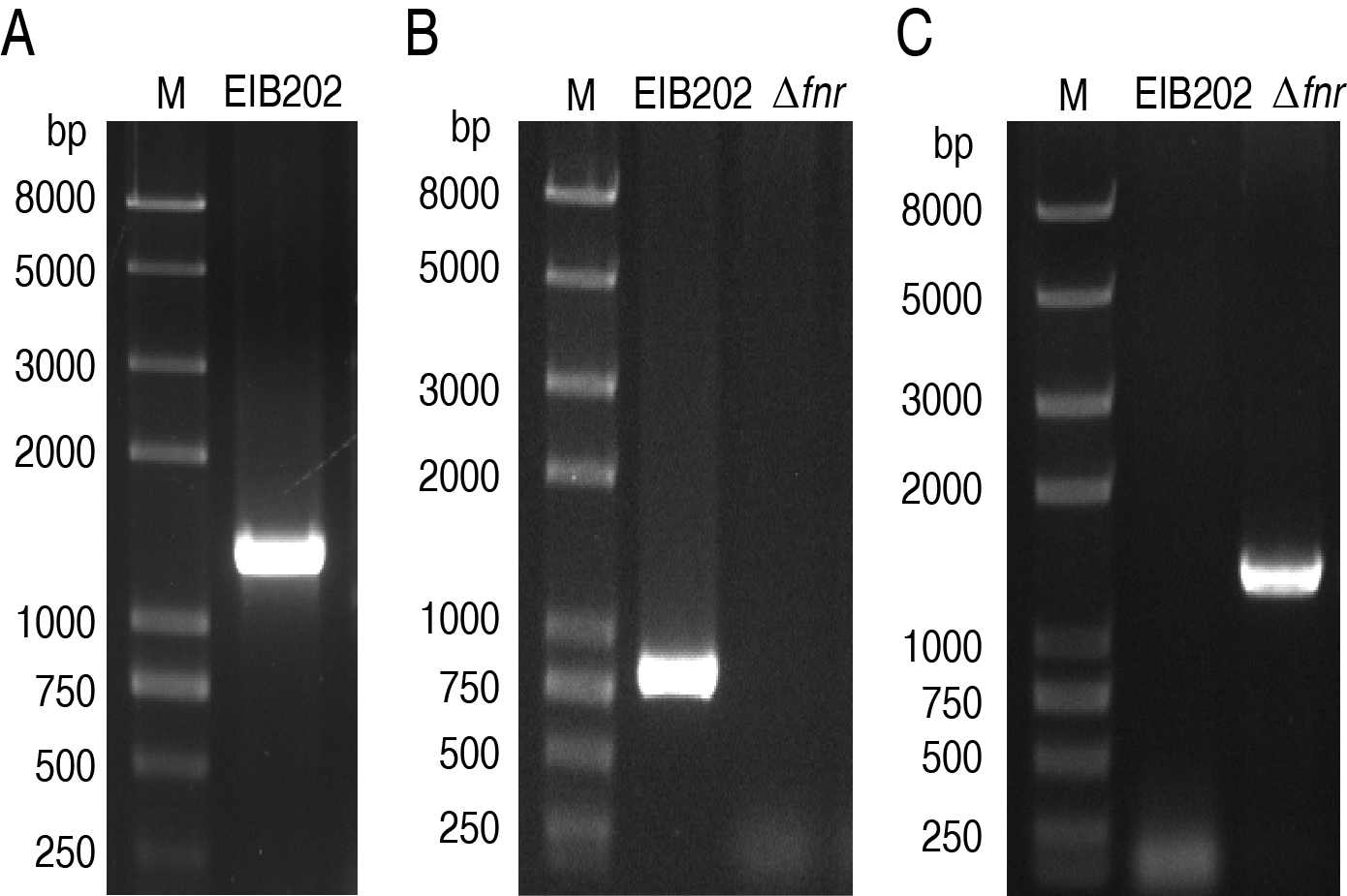


**SUPPLEMENTARY FIGURE S1. Construction of genetically modified strain of *fnr* deletion**

(A) PCR amplification of recombinant fragment from pKD13 using a pair of primer *fnr*-1F and *fnr*-1R.

(B) and (C) Identification of Δ*fnr* by PCR using a pair of primers Kan-F/Kan-R (B) and *fnr*-2F and *fnr*-2R (C).


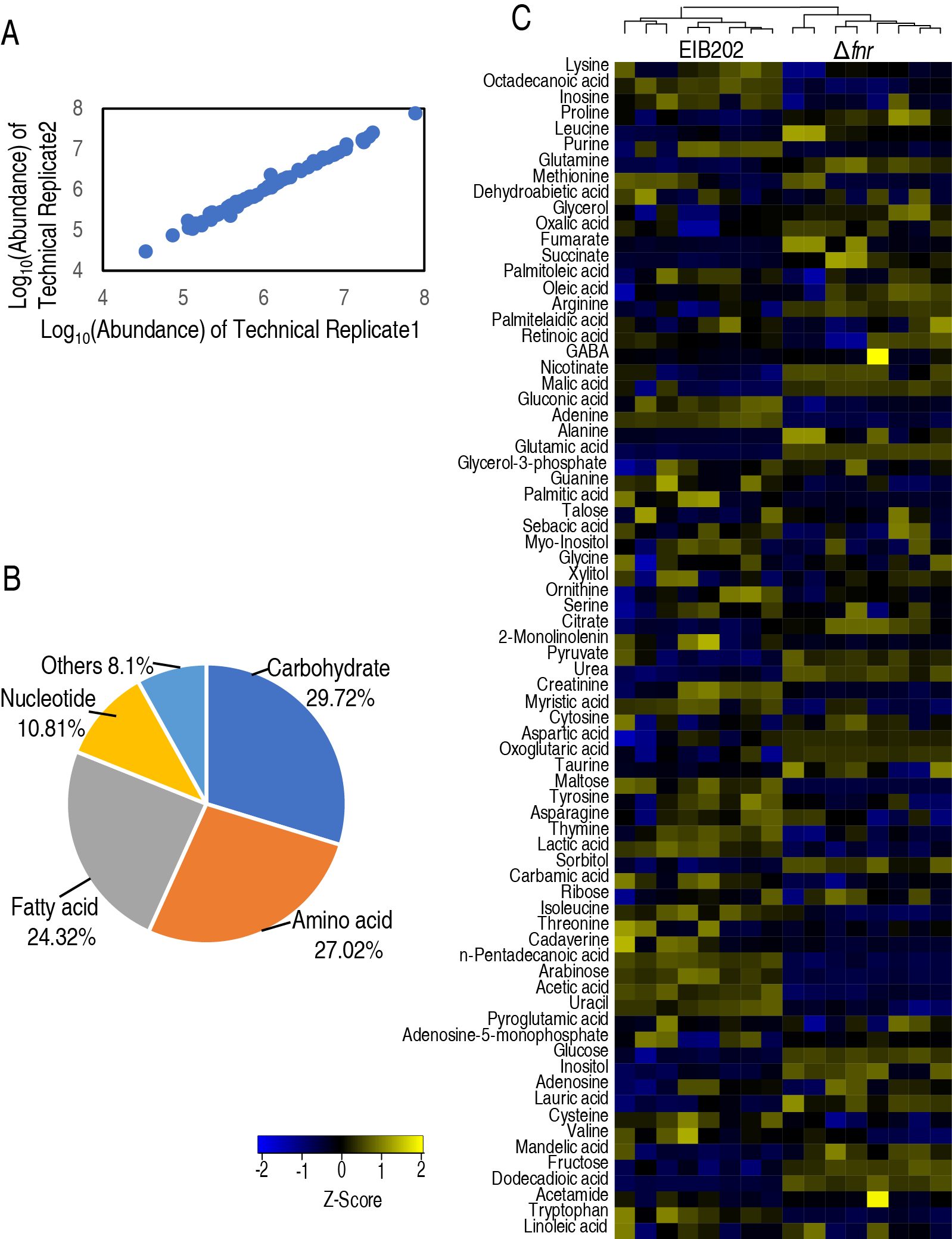


**SUPPLEMENTARY FIGURE 2.**  **Metabolic profiles of Δ*fnr***

**(A)** Reproducibility of the metabolomics profiling platform used in the discovery phase. Metabolite abundances quantified in samples over two technical replicates are shown. The correlation coefficient between technical replicates varies between 0.998 and 0.999. This plot shows the two replicates with the weakest correlation of 0.998.

**(B)** Category of detected metabolites.

**(C)** Unsupervised hierarchical clustering of all metabolites (row). Yellow and blue indicate an increase and decrease of metabolites relative to the mean and standard deviation of the row metabolite level, respectively (see color scale). EIB202 and Δ*fnr* labels at the top indicate the category groupings.

Supplementary Table 1. Primers used for qPCR

| Gene | Primer | Primer sequence |
| --- | --- | --- |
| 16S RNA | Forward | 5'-ACTGAGACACGGTCCAGACTCCTAC-3' |
|  | Reverse | 5'-TTAACGTTCACACCTTCCTCCCTAC-3' |
| *pcK* | Forward | 5'-GATGAACGGAGCCAAATGCA-3' |
|  | Reverse | 5'-GGCCAAAGAAGATCGCCAC-3' |
| *pykF* | Forward | 5'-TTCACCACCGACCAGAGCG-3' |
|  | Reverse | 5'-TCAGGCCATCGTCCACCAG-3' |
| *aceE* | Forward | 5'-ACCTCTGGACGCACCACCCT-3' |
|  | Reverse | 5'-AGTACACGTTCTCCTGCTTCTCA-3' |
| *aceF* | Forward | 5'-CAGGGCGGCTGCTTCACTA-3' |
|  | Reverse | 5'-CGCAAACTCTTTCCCATTCCA-3' |
| *gltA* | Forward | 5'-TGAGCTGGGTATGAATGACGA-3' |
|  | Reverse | 5'-CAGTATGATGCCGGAGTAGAAG-3' |
| *acnB* | Forward | 5'-TCCCTGTGCATGGGCAACC-3' |
|  | Reverse | 5'-CCCACCTTCGCCATAAACTGC-3' |
| *icd* | Forward | 5'-AAACGCCGAGGATATTTACGC-3' |
|  | Reverse | 5'-ATGCCGCAGTGCTCAGGGA-3' |
| *sucA* | Forward | 5'-ACCTACTGCGGAACCATCGG-3' |
|  | Reverse | 5'-TTCTCCTGCGGGCTAAACG-3' |
| *sucC* | Forward | 5'-GCCGAACAGTGGCTGGGTA-3' |
|  | Reverse | 5'-GTCGCTCCCTCCACCAGAAT-3' |
| *sdhA* | Forward | 5'-ACCCGTTCCCATACCGTTTC-3' |
|  | Reverse | 5'-TGCCATTCCCAGTTGTCTTCAT-3' |
| *frdA* | Forward | 5'-GCTGTCCGTGGAGCCGTAA-3' |
|  | Reverse | 5'-CAAACCAGGTGCGTTCAATCT-3' |
| *fumC* | Forward | 5'-GCCGTATGGACTCCTATGTCG-3' |
|  | Reverse | 5'-TCAGGCTCTTGATGCTCTGC-3' |
| *mdh* | Forward | 5'-ATCCGCTCCAACACCTTCG-3' |
|  | Reverse | 5'-CGGCCTTAGCCTCTACCACC-3' |
| *aspC* | Forward | 5’- GCTGTTCCACGGCTGTTGC-3’ |
|  | Reverse | 5’-CAAAGCCCTGATAGGCGAAGT-3’ |
| *ppc* | Forward | 5’-TCGCCAAGTCCGATCTGTG-3’ |
|  | Reverse | 5’- CCGCCTGAATATCTCGCTGTA-3’ |
